# Short-term effects of oral curcumin as adjunct to intravitreal bevacizumab in diabetic macular edema: a randomized clinical trial

**DOI:** 10.1186/s40942-026-00911-5

**Published:** 2026-08-03

**Authors:** Alireza Dehghani, Abbas Abdali, Mohammad Malekahmadi, Elaheh Foroughi, Mahsa Rafiee Alhossaini, Ali Mohammad Sabzghabaee, Mohsen Pourazizi

**Affiliations:** 1 Isfahan Eye Research Center, Department of Ophthalmology, Isfahan University of Medical Sciences, Isfahan, Iran; 2 Student Research Committee, Isfahan University of Medical Sciences, Isfahan, Iran; 3 Isfahan Clinical Toxicology Research Center, Isfahan University of Medical Sciences, Isfahan, Iran

**Keywords:** Diabetic macular edema, Curcumin, Diabetic retinopathy, Bevacizumab, Diabetes

## Abstract

**Purpose:**

To evaluate the efficacy of combined oral curcumin and intravitreal bevacizumab (IVB) in diabetic macular edema (DME).

**Methods:**

This clinical trial was conducted using a parallel-group, randomized, placebo-controlled design in patients with DME. All participants received IVB at the standard dose at monthly intervals for a total of three doses. Additionally, they were assigned to receive either oral curcumin capsules (40 mg) or a matched placebo every 12 h for three months, starting at the time of the first IVB injection. Treatment efficacy was assessed by comparing changes in foveal, perifoveal, and parafoveal retinal thickness, central macular volume (CMV), and LogMAR-corrected distance visual acuity (CDVA). Hemoglobin A1c (HbA1c) tests were performed for all patients at baseline and at three months.

**Results:**

Fifty-two eyes of 52 patients (26 eyes per group) completed the study. The patients’ baseline characteristics were comparable between the groups. Macular thickness and CMV decreased significantly from baseline in both groups, with no statistically significant difference observed between the groups. Similarly, changes in LogMAR CDVA did not differ significantly between the intervention and control groups (MD: 0.08, [95% CI: -0.04 to 0.20]; *P* = 0.12). Both groups demonstrated a significant reduction in HbA1c levels at three months compared to baseline; however, the difference in HbA1c changes between the groups was statistically significant (*P* < 0.001), favoring the curcumin group (MD: 0.63%, 95% CI: [0.3-1.0]). No specific systemic or ocular adverse effects were observed in either group during the study.

**Conclusion:**

Adjunctive treatment with oral curcumin in patients receiving IVB for DME was associated with a significantly greater reduction in HbA1c levels. Despite this metabolic outcome, oral curcumin did not provide additional detectable anatomical or functional benefit over intravitreal bevacizumab alone during the three-month follow-up.

## Introduction

Diabetic macular edema (DME) is a major cause of vision loss in patients with diabetic retinopathy [1]. Understanding the pathophysiological pathways involved in the development of DME is crucial for identifying appropriate treatment. Inflammatory factors and vascular pathways play a role in the development of macular edema [2]. Additionally, elevated peripheral glucose causes oxidative stress and inflammation in the retina. These underlying mechanisms mean that vascular endothelial growth factor (VEGF) is only one part of the disease pathogenesis pathway [3].

Current routine treatments for DME include intravitreal injection of anti-VEGF agents, corticosteroids, fundus laser photocoagulation, and vitreous surgery [4]. Despite a significant reduction in vision loss caused by DME with the adoption of routine treatments, there are limitations, including irreversible damage to the retina from laser photocoagulation and a lack of response to anti-VEGF therapy in approximately 25% of patients. Furthermore, there is a risk of endophthalmitis due to the need for frequent anti-VEGF injections because of their short half-life [5]. These factors have drawn researchers’ attention to new and combination drugs for the treatment of DME.

Curcumin, as the main active component of turmeric (*Curcuma longa*), has been shown to exhibit antioxidant, anti-angiogenesis, anti-inflammatory, and therapeutic potentials, attracting researchers’ attention for supplement therapy in treating various diseases [6–8]. Curcumin acts by scavenging reactive oxygen species and inhibiting the production of pro-inflammatory cytokines [9].

Recently, curcumin has been introduced as a promising therapeutic option for the treatment of retinal diseases and macular edema [10–13]. Previous studies suggest a beneficial effect of curcumin on glycemic control, including significant reduction of fasting blood glucose and hemoglobin A1c (HbA1c) [14–17]. Findings from experimental studies have also supported its efficacy for diabetic retinopathy [18]. However, the clinical application of curcumin in human models has been challenged due to its low bioavailability [19, 20]. Rohillaa et al. 2023 [10] reviewed the therapeutic potentials of curcumin as a new hope in DME. The results of this study were debatable due to the limited clinical intervention on the curcumin efficacy and safety for DME. In this study, we investigate the effect of combining oral curcumin with intravitreal bevacizumab (IVB) compared to bevacizumab monotherapy through a randomized, double-blind clinical trial in treating DME.

## Methods

### Study design

This clinical trial was conducted using a 3-month, parallel-group, randomized, double blind, placebo-controlled design in patients with DME in the retina clinic of Feiz Hospital, affiliated with Isfahan University of Medical Sciences, from June 2023 to June 2024. This study was conducted in accordance with the Declaration of Helsinki and was approved by the Ethics Committee of the Isfahan University of Medical Sciences (IR.MUI.REC.1402.336). The research protocol is also available on ClinicalTrials.gov (NCT06595355, 2024-09). Written informed consent was obtained from all the enrolled patients.

### Participants

Patients aged at least 40 years with type 2 diabetes were eligible if they were diagnosed with center-involving macular edema (CI-DME) on Optical Coherence Tomography (OCT), demonstrated by a central macular thickness (CMT) greater than 320 μm in males and 305 μm in females [21]. Patients were required not to have received an intravitreal injection or any type of laser photocoagulation within the last three months before the intervention. The patients enrolled in this trial had regular diet, which would be expected to minimize potential dietary-related differences between the study groups.

Patients with any of the following were not included: [1] a history of other retinal diseases or macular edema due to other causes such as uveitis, epiretinal membrane, or central retinal vein occlusion; [2] the presence of proliferative diabetic retinopathy with or without vitreous hemorrhage, or a history of vitrectomy; [3] glaucoma requiring intraocular pressure-lowering medication, concurrent vitreous hemorrhage, or age-related macular degeneration; [4] significant media opacities limiting the interpretation of diagnostic tests; [5] any ocular surgery within the 6 months before the baseline visit; [6] current pregnancy or lactation; [7] a known history of allergy to curcumin; or [8] any systemic disease requiring ongoing corticosteroid therapy.

Furthermore, patients were excluded from the study if there was a change in their clinical diagnosis during the study course or if they required surgical intervention, any significant changes in the patient’s general health status, failure to attend follow-up visits, inability to complete follow-up, or patient withdrawal of consent for continued treatment and monitoring.

Only one eye per participant was included in the study. If both eyes met the inclusion criteria, the eye with the greater CMT was selected.

### Intervention arms

All participants received intravitreal Stivant^®^ (CinnaGen Co., Tehran, Iran), which emerges as a biosimilar to bevacizumab at the standard dose of 1.25 mg/0.05 ml at monthly intervals for a total of three doses. Additionally, they were assigned to receive either an oral SinaCurcumin^®^ soft gel capsule (40 mg curcuminoids per capsule, manufactured by Exir Nano Sina Company and marketed by Sinadarou Pharmaceutical Company, Tehran, Iran) or a matched placebo every 12 h for three months, starting from the time of the first IVB injection. SinaCurcumin^®^ is a nanomicellar formulation designed to improve the solubility and oral bioavailability of curcumin significantly. The placebo capsules were identical to the active SinaCurcumin^®^ capsules in appearance, size, color, texture, and odor to maintain blinding. Patients were randomly assigned to one of two groups: the intervention group (IVB + Curcumin) and the control group (IVB + Placebo).

Dosette boxes were supplied to participants monthly to enhance medication adherence. Compliance was monitored through patient self-reports and by counting the capsules returned at each visit.

### Sample size

Enrolling 26 participants in each group was deemed sufficient to detect a between-group effect size (d) of 0.93 using a 2-tailed t-test of mean differences, assuming 95% power, 5% significance level, and based on the methodology reported by Parravano et al. [19].

### Randomization and masking

Independent individuals were responsible for randomization and masking procedures. Eligible participants were randomly assigned in a 1:1 ratio using Random Allocation Software. The identical appearance, texture, and sensory characteristics of curcumin and placebo capsules maintained the masking of participants, care providers, and outcome assessors.

### Tools and assessments

After patient selection, all patients underwent ophthalmic examinations at the study baseline and then monthly for three months. Ocular adverse events were evaluated with the assessment of VA using a Snellen chart at a distance of 20 feet, slit-lamp biomicroscopic examination of the anterior segment, detailed funduscopic examination, and tonometry. Also, complications related to bevacizumab injection were examined in the patients. Systemic adverse events, including gastrointestinal discomfort, drug intolerance, allergy, and others, were evaluated by questioning the patients.

Participants underwent optical coherence tomography (OCT) imaging of the macula, using a SPECTRALIS OCT system, Spectral-Domain OCT (Spectralis^®^, Heidelberg Engineering, Heidelberg, Germany). Foveal, perifoveal, and parafoveal retinal thickness and central macular volume (CMV) were measured in all patients at baseline and one month after the last injection. Additionally, HbA1c tests were performed for all patients at baseline and three months later. Visual acuity (VA) was transformed into the logarithm of the minimum angle of resolution (LogMAR).

### Outcomes

The primary outcomes were group differences in macular thickness (MT) and LogMAR VA from baseline to endpoint. HbA1c changes, CMV changes, and frequency of side effects between the groups were the secondary outcomes.

### Statistical analysis

The Statistical Package of Social Science Software 21 (IBM, Armonk, NY, USA) was used for statistical analyses. A significance level of 0.05 was used. The normality of the data was assessed using the Shapiro–Wilk test and visual inspection of Q–Q probability plots.

Descriptive variables are presented as mean and standard deviation (SD) for continuous and number (percentage) for categorical variables. Group comparisons were conducted using the independent samples t-test or Mann–Whitney U test for continuous variables and the Chi-square test or Fisher’s exact test for categorical variables, as appropriate. Within-group changes were analyzed using the paired samples t-test or the Wilcoxon signed-rank test. Between-group comparisons at follow-up were performed using analysis of covariance (ANCOVA) adjusted for baseline values.

## Results

### Study participants

**Fig. 1:**
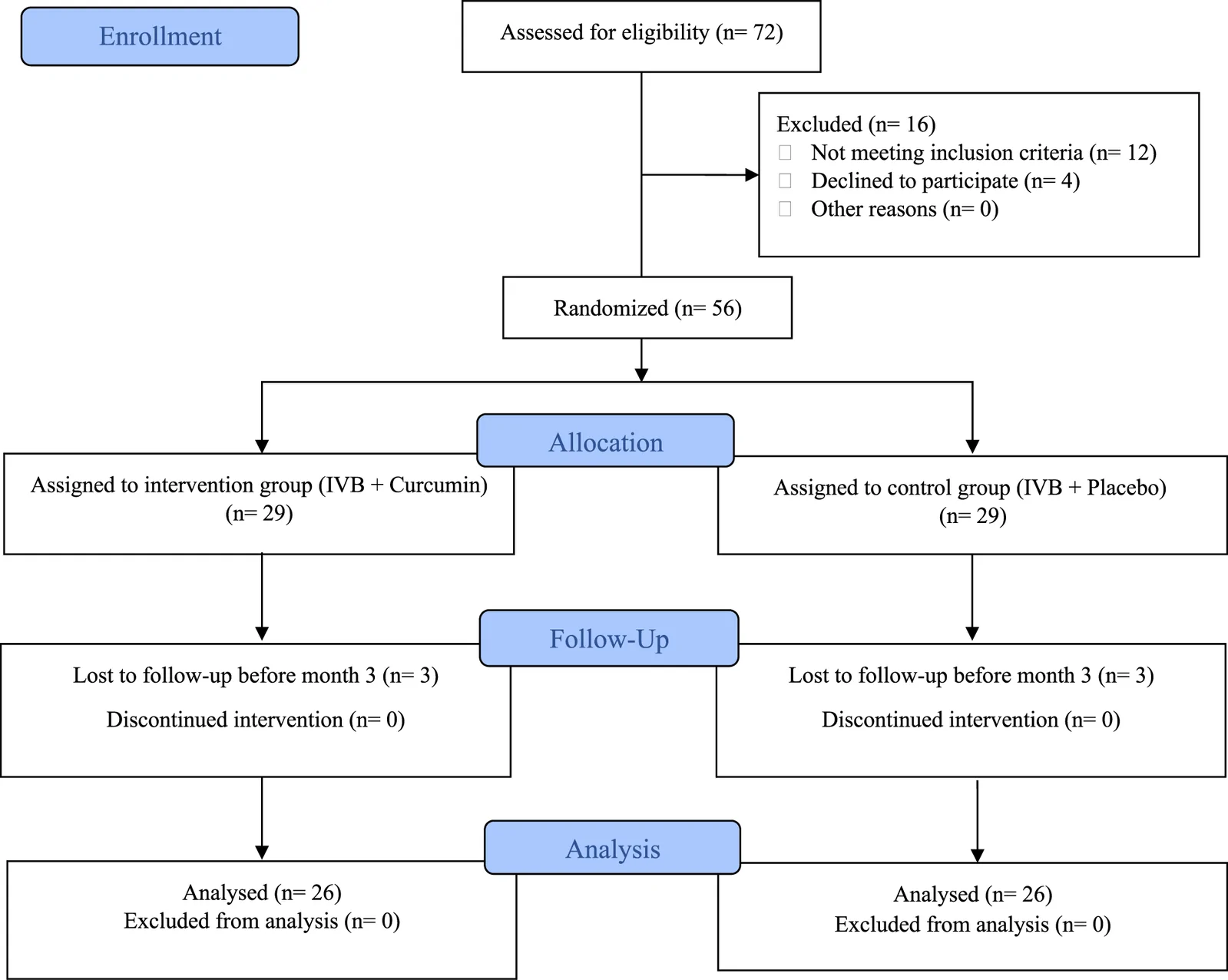
CONSORT flow diagram

The flow diagram of subject enrollment to data analysis is presented in Fig. 1. Fifty-two eyes with DME from 52 patients were enrolled in this randomized clinical trial.

The patients’ baseline demographic and clinical characteristics are reported in Table 1.

**Table 1:** Baseline demographics and ocular characteristics of the patients

Variable	IVB+Curcumin Group (*n* = 26 eyes)	IVB+Placebo Group (*n* = 26 eyes)	*P*-Value
**Age**,** year Mean ± SD**	62.8 ± 8.5	66.2 ± 9.5	0.18^a^
**Sex**,** N (%)**			0.40^b^
Female	10 (38.5%)	13 (50.0%)	
Male	16 (61.5%)	13 (50.0%)	
**Duration of DM**,** Mean year ± SD**	12.5 ± 4.5	12.7 ± 5.3	0.86^a^
**Laterality**,** N (%)**			0.57^b^
OD	11 42.3(%)	9 (34.6%)	
OS	15 (57.7%)	17 (65.4%)	
**Treatment type**,** N (%)**			1.000^c^
INS	23 (88.5%)	22 (84.6%)	
TAB	3 (11.5%)	4 (15.4%)	
**HbA1c (%)**	8.0 ± 1.2	8.0 ± 1.0	0.91^d^
**CMT (µm)**	446.2 ± 117.9	423.9 ± 91.9	0.45^a^
**CMV (mm** ^**3**^ **)**	10.7 ± 2.2	10.2 ± 1.3	0.56^d^
**LogMAR VA**	0.7 ± 0.3	0.7 ± 0.3	0.69^d^

Abbreviations: SD: Standard deviation; DM: Diabetes mellitus; OD: Right eye; OS: Left eye; IVB: Intravitreal Bevacizumab; CMT: Central macular thickness; LogMAR VA: Logarithm of the minimum angle of resolution visual acuity; CMV: Central macular volume; N: Number; HbA1c: Hemoglobin A1; INS: Insulin therapy; TAB: Oral antidiabetic tabletsa: independent samples t-test; b: Chi-square test; c: Fisher’s exact test; d: Mann-Whitney test

### Demographic and baseline characteristics

No significant differences were observed in the demographic and baseline characteristics between the two groups (Table 1). Primary diabetic medications were adjusted between the two groups (p-value: 1.000), and systemic anti-diabetic medications remained unchanged in dose and type during the three-month study period in both groups. Additionally, ocular parameters at baseline, including MT, LogMAR VA, and CMV, also showed no significant differences between groups (all *p* > 0.05), indicating successful randomization.

### Follow-up outcomes

#### Macular thickness and volume

**Fig. 2:**
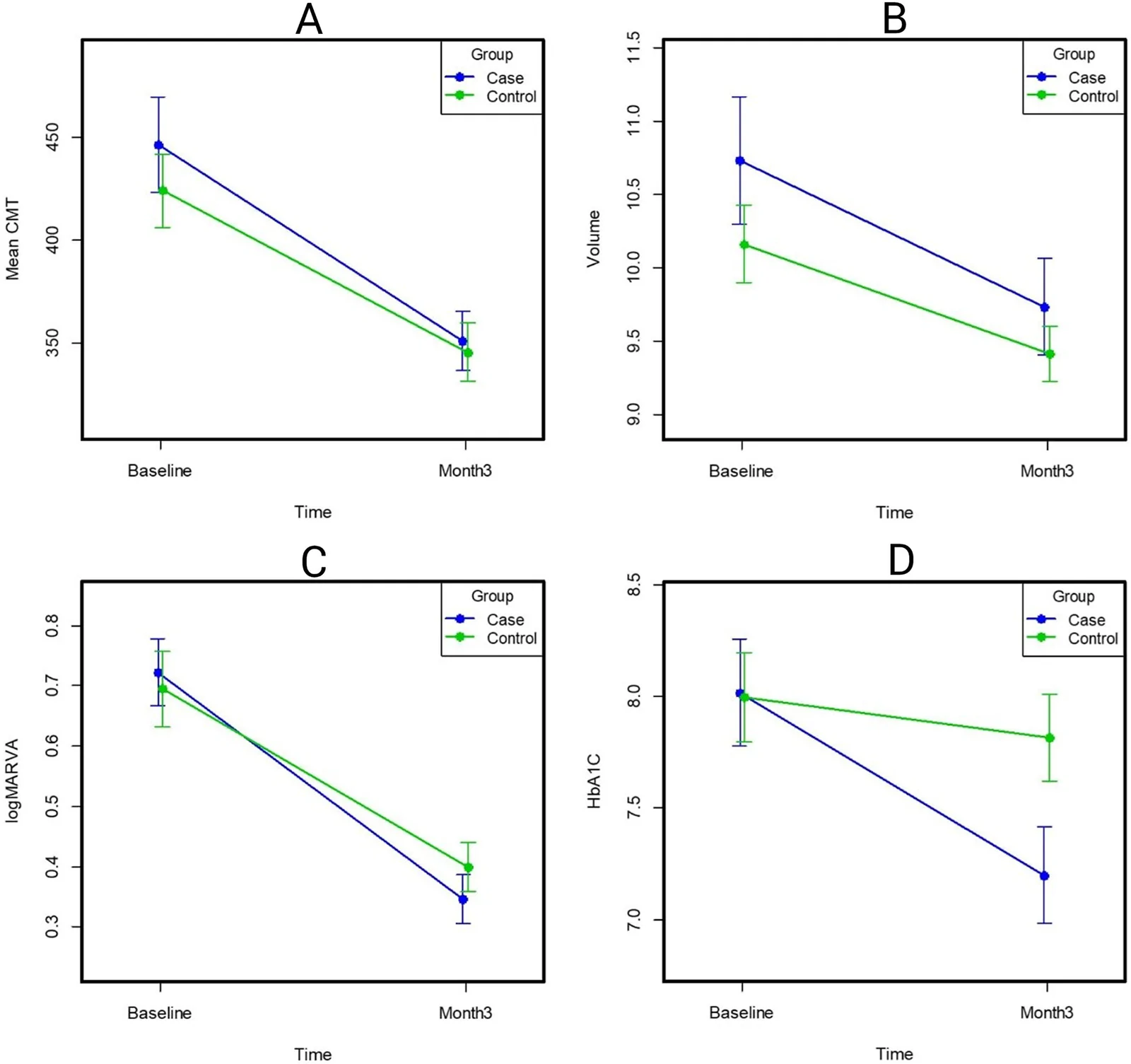
Longitudinal Changes in measurement outcomes from baseline to month 3 in the case and control groups. (**A**) Central macular thickness (**B**) Central macular volume (**C**) LogMAR visual acuity. (**D**) Hemoglobin A1C

CMT was significantly reduced in both groups (mean change: 95.7 ± 99.1 μm, *P* < 0.001 for the intervention group; 78.5 ± 86.6 μm, *P* < 0.001 for the control group). No significant between-group difference was observed (*P* = 0.88) (Fig. 2-A). All measured parafoveal and perifoveal sectors (superior, temporal, inferior, nasal) showed significant within-group reductions in both groups (all *P* < 0.01). But similar to CMT, no significant difference was observed between the intervention and control groups (P-values ranging from 0.34 to 0.96) (Table 2). The macular volume (MV) significant reductions also occurred in both groups (mean change: 0.1 ± 1.1 mm³, *P* < 0.001 in the IVB+Curcumin group, and 0.8 ± 0.7 mm³, *P* < 0.001 in the IVB+Placebo group). But the between-group difference was not significant (*P* = 0.77) (Table 2) (Fig. 2-B).

**Table 2:** Comparison of the pre- and post-intervention measures changes in the case and control group

Parameter	Time	Group		Diff.^**^	95% CI^€^		*P* ^†^
		Case	Control		Lower	Upper	
HbA1C (%)	Baseline	8.0 ± 1.2	8.0 ± 1.0	0.02	-0.6	0.6	-
	Month 3	7.2 ± 1.1	7.8 ± 1.0	-0.61	-1.2	-0.03	**< 0.001**
	Change*	0.8 ± 0.8	0.2 ± 0.3	0.63	0.3	1.0	
	P-within^¥^	**< 0.001** ^a^	**0.005** ^a^				
CMT (µm)	Baseline	446.2 ± 117.9	423.9 ± 91.9	22.35	-36.5	81.2	-
	Month 3	350.6 ± 73.6	345.4 ± 73.2	5.23	-35.7	46.1	0.88
	Change	95.7 ± 99.1	78.5 ± 86.6	17.11	-34.7	69.0	
	P-within	**< 0.001** ^a^	**< 0.001** ^a^				
LogMAR VA	Baseline	0.7 ± 0.3	0.7 ± 0.3	0.03	-0.1	0.2	-
	Month 3	0.4 ± 0.2	0.4 ± 0.2	-0.05	− 0.02	0.06	0.12
	Change	0.4 ± 0.2	0.3 ± 0.2	0.08	-0.04	0.2	
	P-within	**< 0.001** ^b^	**< 0.001** ^b^				
Macular volume (mm^3^)	Baseline	10.7 ± 2.2	10.2 ± 1.4	0.57	-0.5	1.6	-
	Month 3	9.7 ± 1.7	9.4 ± 1.0	0.32	-0.4	1.1	0.77
	Change	1.0 ± 1.1	0.8 ± 0.7	0.25	-0.3	0.8	
	P-within	**< 0.001** ^b^	**< 0.001** ^a^				
Perifoveal superior thickness (µm)	Baseline	365.4 ± 96.6	341.46 ± 48.60	23.92	-19.1	66.9	-
	Month 3	348.4 ± 82.7	329.0 ± 45.2	19.35	-18.0	56.7	0.88
	Change	17.0 ± 48.4	12.4 ± 27.9	4.58	-17.4	26.6	
	P-within	**0.004** ^b^	**0.004** ^b^				
Perifoveal temporal thickness (µm)	Baseline	371.9 ± 94.3	350.2 ± 65.5	21.73	-23.5	67.0	-
	Month 3	345.7 ± 89.6	333.9 ± 66.8	11.85	-32.2	55.9	0.34^‡^
	Change	26.2 ± 47.9	16.4 ± 27.4	9.88	-11.9	31.6	
	P-within	**0.001** ^b^	**0.006** ^b^				
Perifoveal inferior thickness (µm)	Baseline	353.5 ± 80.6	337.0 ± 56.2	16.50	-22.2	55.2	-
	Month 3	331.4 ± 74.5	310.42 ± 30.99	20.96	-11.2	53.2	0.35^†^
	Change	22.1 ± 37.2	26.5 ± 35.4	-4.46	-24.7	15.7	
	P-within	**0.001** ^b^	**0.001** ^a^				
Perifoveal nasal thickness	Baseline	352.4 ± 49.4	349.0 ± 48.4	3.42	-23.8	30.7	-
	Month 3	332.2 ± 37.2	330.0 ± 31.1	2.23	-16.9	21.3	0.96^†^
	Change	20.2 ± 32.5	19.0 ± 23.7	1.19	-14.6	17.0	
	P-within	**0.002** ^b^	**< 0.001** ^a^				
Parafoveal superior thickness (µm)	Baseline	433.2 ± 96.5	409.9 ± 71.1	23.35	-23.9	70.6	-
	Month 3	381.7 ± 62.9	375.4 ± 55.5	6.27	-26.8	39.3	0.46^†^
	Change	51.5 ± 59.0	34.5 ± 37.5	17.08	-10.5	44.6	
	P-within	**< 0.001** ^a^	**< 0.001** ^a^				
Parafoveal temporal thickness (µm)	Baseline	447.9 ± 105.9	415.8 ± 83.9	32.15	-21.1	85.4	-
	Month 3	385.9 ± 77.8	383.4 ± 67.6	2.46	-38.1	43.1	0.26^†^
	Change	62.0 ± 70.6	32.4 ± 56.6	29.69	-6.0	65.3	
	P-within	**< 0.001** ^b^	**0.007** ^a^				
Parafoveal inferior thickness (µm)	Baseline	424.9 ± 90.7	404.0 ± 67.5	20.88	-23.7	65.4	-
	Month 3	377.1 ± 61.2	359.5 ± 36.5	17.62	-10.4	45.8	0.41^†^
	Change	47.8 ± 52.8	44.5 ± 49.9	3.27	-25.4	31.9	
	P-within	**< 0.001** ^b^	**< 0.001** ^a^				
Parafoveal nasal thickness (µm)	Baseline	417.9 ± 74.4	400.1 ± 60.1	17.77	-20.4	55.9	-
	Month 3	367.9 ± 42.1	359.5 ± 38.1	8.46	-13.9	30.8	0.89^†^
	Change	49.9 ± 59.7	40.7 ± 35.8	9.31	-18.1	36.8	
	P-within	**< 0.001** ^a^	**< 0.001** ^a^				

*Change: Baseline- Month3, ^¥^ Within group p-value based on paired sample test (^a^t-test or ^b^Wilcoxon signed ranks test), ^†^ Between-group p-value, based on ANCOVA model adjusted for the baseline, ^‡^Between-group p-value based on ANOVA model for relative difference, **Diff.: Mean difference (Case-Control), ^**€**^ 95% CI: [Mean difference ± Standard error × 1.96]; CMT: Central macular thickness; LogMAR VA: Logarithm of the minimum angle of resolution visual acuity; HbA1c: Hemoglobin A1

### Visual acuity

LogMAR VA improved significantly in both groups compared to pre-intervention values. However, no significant difference was observed between the two groups (*P* = 0.12) (Table 2) (Fig. 2-C).

### HbA1c levels

At the 3-month follow-up, HbA1c levels were significantly reduced compared to baseline values within both groups (mean change: 0.8% ± 0. 8%, *P* < 0.001 for the curcumin group; and 0.2% ± 0.3%, *P* = 0.005 for the placebo group). However, the difference in HbA1c changes between the groups was statistically significant (*P* < 0.001), favoring the curcumin group (MD: 0.63%, 95% CI: [0.3-1.0])(Table 2) (Fig. 2-D).

### Adverse effects and adherence to the treatment protocol

During the three-month follow-up period, no ocular or systemic adverse event that affected the results and safety of the study was detected. All patients tolerated the treatments without significant problems. During frequent clinic visits and ongoing follow-up, patients’ adherence to the prescribed regimen was satisfactory.

## Discussion

In this randomized, placebo-controlled trial, the primary outcomes were between-group differences in macular thickness and LogMAR at the 3-month follow-up. The observed greater reduction in HbA1c levels in the curcumin group compared to the control group was associated with adjunctive curcumin therapy and may suggest a systemic biological effect.

Although both treatment groups showed significant improvement in all anatomical and functional parameters from baseline, no statistically significant differences were observed between the curcumin and placebo groups in any of the primary ophthalmic outcomes. These findings demonstrate that adjunctive oral curcumin did not provide additional detectable short-term anatomical or functional benefit over intravitreal bevacizumab monotherapy in patients with center-involving diabetic macular edema.

The significant reduction of HbA1c in both the placebo and intervention groups was achieved. The reduction was significantly higher in the curcumin treatment group than in the placebo group. A significant decrease in HbA1c in both groups can reflect better general diabetic care. Participation in clinical studies often leads to a more serious adherence to medical treatments and diet regimen.

Given the diverse morphology of DME and the variable clinical response to standard anti-VEGF therapy, investigating the underlying pathophysiology is essential for developing new treatment strategies. Hyperglycemia is the strongest risk factor for the development of DME. Hyperglycemia leads to increased oxidative stress, inflammation, and vascular dysfunction through various pathways. Oxidative stress and inflammation drive the upregulation of growth factors and cytokines such as VEGF, angiopoietins, tumor necrosis factor-alpha (TNF-α), interleukins (IL-1β, IL-6, IL-8), chemokines (MCP-1), and matrix metalloproteinases (MMP-2, MMP-9), all of which contribute to the breakdown of the blood-retinal barrier (BRB) and subsequent diabetic macular edema [2, 22–24]. Systemic, oral, or topical therapies with broad anti-inflammatory mechanisms (such as curcumin, corticosteroids, fenofibrate, TNF-α inhibitors, or IL-6 inhibitors) could have a complementary or even alternative role [2, 25].

Curcumin, a natural polyphenolic compound extracted from turmeric (*Curcuma longa*) with known anti-inflammatory properties, has shown promising results in the formulation of nutraceuticals for the treatment of eye diseases. Curcumin can exert its anti-inflammatory effects by reducing the release of pro-inflammatory cytokines and inhibiting the activation of inflammatory pathways. It also reduces oxidative stress and protects retinal cells through various pathophysiological pathways. In addition, previous studies have suggested its protective effects on the blood-retinal barrier, reduced retinal blood flow, and reduced VEGF production [10, 12].

Although curcumin may naturally be present in routine diets, particularly in Asian populations, the amount of dietary curcumin intake varies among individuals depending on their nutritional habits. Therefore, the use of standardized supplementation may reduce the potential confounding effect of dietary curcumin exposure. In the present study, patients with a regular diet were enrolled, and participants were randomized into the intervention and placebo groups. Accordingly, considering the comparability between the two groups following randomization, the observed differences in outcomes are more likely attributable to curcumin supplementation.

In the study by Ferrara et al. 2020 [11] Curcumin-based treatment in patients with macular edema of uncommon etiologies resulted in improved CDVA and reduced CMT after 6 months of follow-up, with no adverse effects reported. Additionally, in another study, treatment of DME using curcumin yielded favorable results after three months [13].

In this study, HbA1c in the curcumin group showed a significantly greater reduction than in the control group at 3-month follow-up. This may indicate the systemic effects of curcumin on metabolic pathways and systemic inflammation. In contrast, the OCT findings and VA did not differ. This lack of difference may be due to several factors. Given that curcumin’s effects are likely gradual and cumulative, and its oral bioavailability is limited, the three-month follow-up period may have been too short to detect significant structural changes. The multiple pathways involved in the underlying pathophysiology of DME and the role of curcumin in modulating these pathways can also strengthen this hypothesis. Furthermore, in routine clinical practice, intravitreal bevacizumab for diabetic macular edema is commonly initiated with monthly loading injections followed by reassessment of retinal response to treatment. Therefore, a 3-month follow-up period was considered appropriate for evaluating the early therapeutic response in the present study [26]. Additionally, the potent therapeutic effect of intravitreal bevacizumab in CI-DME may have masked the subtle adjunctive effects of curcumin supplementation.

Although the addition of oral curcumin was associated with a significantly greater reduction in HbA1c in the intervention group compared to the control group, the primary aim of this study was to evaluate the structural and functional ophthalmic effects of adjunctive curcumin therapy. Moreover, detailed information regarding dietary habits, physical activity, medication adherence, and other lifestyle factors was not collected. Therefore, we cannot exclude the possibility of residual confounding, and causality between curcumin supplementation and the observed HbA1c reduction cannot be firmly established.

Overall, the initial results are promising, but larger, multicenter studies investigating different doses, higher bioavailability formulations, longer follow-up duration, and different patient subgroups based on metabolic control are needed to definitively confirm the effects of curcumin in DME. Additionally, subgroup analysis based on edema morphology and structural variables was not performed in the present study because of the limited sample size. This may have underpowered the study to detect subtle adjunctive effects, as the sample size was calculated based on a large expected effect size, whereas a systemic therapy added to anti-VEGF is likely to have a smaller effect. Therefore, the absence of significant between-group differences should be interpreted cautiously and does not definitively exclude a modest benefit.

Although baseline characteristics were comparable between the two groups, we did not stratify patients according to prior DME treatment history (treatment-naïve versus previously treated, refractory, or recurrent cases), which may influence the results. Given that previous anti-VEGF exposure can significantly influence the response to subsequent injections, the lack of such stratification represents a limitation. Additionally, quantitative assessment of treatment adherence was not formally recorded for the intervention and control groups. Future studies are recommended to perform subgroup analyses according to the treatment history to eliminate this potential confounder and better evaluate the efficacy of adjunctive curcumin in different patients. The effect of curcumin in different morphologic patterns of DME could also be considered in future studies.

## Conclusion

Adjunctive curcumin therapy was associated with a significantly greater reduction in HbA1c levels at the three-month follow-up compared with IVB alone. Despite this metabolic outcome, oral curcumin did not provide additional detectable anatomical or functional benefit over intravitreal bevacizumab alone during the three-month follow-up in this study. However, given the limited statistical power to detect modest effects, these findings should not be interpreted as definitive evidence of no benefit. Longer and larger studies are needed to determine whether the observed metabolic effect has any delayed anatomical or functional relevance in DME.

## Data Availability

The data used to support the findings of this study are available from the corresponding author upon request.

## Abbreviations

DME: Diabetic macular edema
DR: Diabetic retinopathy
SD-OCT: Spectral-Domain Optical Coherence Tomography
CMT: Central macular thickness
MV: Macular volume
MD: Mean difference
IL: Interleukin
MMP: Matrix metalloproteinase
MCP: Monocyte chemoattractant protein
VA: Visual acuity
anti-VEGF: Anti-vascular endothelial growth factor
BRB: Blood-retinal barrier
TNF: Tumor necrosis factor
IOP: Intraocular pressure
ETDRS: Early Treatment of Diabetic Retinopathy Study
IVB: Intravitreal Bevacizumab
CDVA: Corrected distance visual acuity
HgA1C: Hemoglobin A1
